# BPF-Based Thermal Sensor Circuit for On-Chip Testing of RF Circuits

**DOI:** 10.3390/s21030805

**Published:** 2021-01-26

**Authors:** Josep Altet, Enrique Barajas, Diego Mateo, Alexandre Billong, Xavier Aragones, Xavier Perpiñà, Ferran Reverter

**Affiliations:** 1 Electronic Engineering Department, Universitat Politècnica de Catalunya–BarcelonaTech, 08034 Barcelona, Spain; enrique.barajas@upc.edu (E.B.); diego.mateo@upc.edu (D.M.); alexandre.billong1@gmail.com (A.B.); xavier.aragones@upc.edu (X.A.); ferran.reverter@upc.edu (F.R.); 2 Institute of Microelectronics of Barcelona (IMB-CNM)—CSIC, 08193 Cerdanyola del Vallés, Spain; xavier.perpinya@imb-cnm.csic.es

**Keywords:** CMOS thermal sensor, CMOS built-in sensor, CMOS integrated circuits, measurement of RF CMOS circuits, built-in test and measurement

## Abstract

A new sensor topology meant to extract figures of merit of radio-frequency analog integrated circuits (RF-ICs) was experimentally validated. Implemented in a standard 0.35 μm complementary metal-oxide-semiconductor (CMOS) technology, it comprised two blocks: a single metal-oxide-semiconductor (MOS) transistor acting as temperature transducer, which was placed near the circuit to monitor, and an active band-pass filter amplifier. For validation purposes, the temperature sensor was integrated with a tuned radio-frequency power amplifier (420 MHz) and MOS transistors acting as controllable dissipating devices. First, using the MOS dissipating devices, the performance and limitations of the different blocks that constitute the temperature sensor were characterized. Second, by using the heterodyne technique (applying two nearby tones) to the power amplifier (PA) and connecting the sensor output voltage to a low-cost AC voltmeter, the PA’s output power and its central frequency were monitored. As a result, this topology resulted in a low-cost approach, with high linearity and sensitivity, for RF-IC testing and variability monitoring.

## 1. Introduction

The temperature in a surface point of an integrated circuit (IC) depends on the power dissipated by the devices placed nearby (so-called self-heating and thermal coupling), the structure and materials that constitute the packaging (which determine the thermal impedances of the different devices) and the ambient temperature [1]. Traditionally, off-chip temperature measurement set-ups have been used to detect unexpected hot spots within digital circuits [2,3,4,5]. Hot spots might appear either due to the presence of a defect in the circuit structure [6,7,8,9] or by a nonuniform power dissipation on the die surface, which is a common situation in microprocessors [4,10]. In complex digital systems, such as microprocessors, temperature sensors are built-in within the same silicon die in order to ensure reliable system performance, i.e., they perform power-temperature monitoring to control the activation of cooling systems, to modulate microprocessor supply voltage or clock frequency, or to assert if the workload of a specific microprocessor block can be increased or should be reduced to avoid nonuniform power distributions [11,12,13,14,15,16,17,18]. Nevertheless, thermal monitoring is not restricted to digital circuits, but used as well in analog circuits. Most commonly, thermal measurements of analog circuits are usually performed to extract the thermal resistance of devices [19,20], especially in power devices. More recently, temperature measurements are done in high frequency analog circuits to perform testing applications. The test of high frequency analog circuits is a challenging task, as the presence of a defect or the effects of process-voltage-temperature variations and device aging, does not usually produce a catastrophic circuit failure, but a degradation of circuit performance and specifications [21,22,23,24,25], which compromises yield. A strategy to compensate these performance degradations is to build in monitor circuits with the analog circuit (called hereafter the circuit under test (CUT)) to track variations in their performance [26,27,28,29,30] and, in the eventual case of detecting a circuit degradation, to activate a feedback in the CUT bias to compensate for them. Recently, several studies have proved that by measuring the temperature in a surface point near the CUT, it is feasible to monitor the performances of radio frequency (RF) and millimeter wave (mmW) circuits [30,31,32,33,34,35,36,37] or the presence of structural defects [38,39]. The use of built-in temperature sensors to monitor high-frequency analog circuits is attractive. On the one hand, the sensor does not load any node of the CUT, avoiding the need of a CUT-sensor codesign. On the other hand, thanks to the Joule effect, there is a frequency down-conversion of the high-frequency information in the electrical domain to low frequency in the thermal domain in such a way that the same temperature sensor can be used to monitor different CUTs working at different frequency bands.

In this scenario, the goal of temperature measurements is to get solely a signature of the power dissipated by the CUT, as this power carries information of the high frequency electrical signals while rejecting any temperature variation due to changes in the ambient temperature or the case-to-ambient thermal resistance (e.g., the activation of a cooling system). To this end, one strategy is the use of differential temperature sensors embedded in the same silicon die [37,38,39,40,41]. Such sensors possess two sensing devices (temperature transducers T1 and T2). Whereas T1 is placed close to the CUT, T2 is placed far from it. This placement ensures that only T1 measures the temperature changes caused by the power dissipation of the CUT, but both T1 and T2 detect common-mode temperature changes, being eventually rejected. Generally, the two sensing devices form the differential pair of an operational transconductance amplifier (OTA), which operates in open-loop to have a high sensitivity [38,39,40]. Although these sensors have been proved useful to perform the test of analog high-frequency CUTs, they have several limitations. Firstly, the OTA output can be easily saturated by mismatches produced because of manufacturing variability and the DC temperature gradients generated by the DC CUT bias [36,38]. Secondly, since the OTA operates in open loop, the gain (and hence the sensitivity to temperature) is quite sensitive to integrated circuit (IC) manufacturing process variations [37]. Thirdly, mismatches, circuit topology and device limitations reduce the rejection to common mode temperature changes.

**Figure 1:**
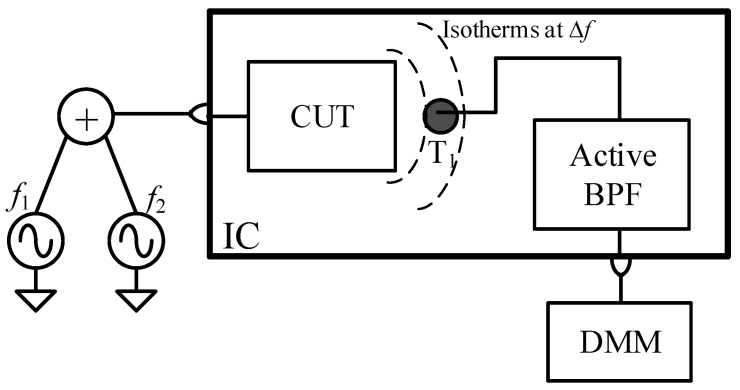
Circuit under test (CUT) with heterodyne driving. Temperature sensor made of a single metal -oxide semiconductor (MOS), transistor temperature transducer (T_1_), an active Band Pass Filter (BPF), and a Digital Multimeter (DMM). Temperature variations at Δ*f* carry information about the CUT figures of merit at *f*_1_.

These limitations have motivated the design of a novel circuit topology that we presented in [42] (Figure 1). One sensing device (T_1_ placed near the CUT) is connected to an active band-pass filter (BPF). The filter’s band-pass is centered at Δ*f* so that the bandwidth (and hence the noise) is limited around the frequency of interest. The BPF’s zero at the origin rejects slow temperature variations (provoked by either ambient temperature changes or the DC CUT power dissipation). To achieve a CUT thermal signature within the frequency band of the sensor, we propose to excite the CUT with a heterodyne technique [41,43]. The technique consists of applying two tones, whose frequencies are *f*_1_ and *f*_2_ = *f*_1_ + Δ*f*, to the CUT input. This driving strategy generates a spectral component of power dissipated by the CUT devices (and hence of temperature) at Δ*f*, whose magnitude depends on the CUT figures of merit at *f*_1_. For Δ*f* values higher than a certain threshold (fixed by both the die thickness and semiconductor thermal properties [40]), the amplitude of this temperature component is no longer dependent on the package materials and package mounting thermal properties, i.e., the silicon die is seen by the CUT, from a thermal point of view, as a semi-infinite medium, independent of the thermal boundary conditions (including ambient temperature). This heterodyne technique is not new: it has been already used with differential temperature sensors connecting the sensor’s output node to a lock-in amplifier (LIA) locked at Δ*f* [33,35]. To reduce costs, the BPF output signal is proposed to be measured using a low-cost digital multimeter (DMM), off-chip in this work, but that allows an easier integration than the LIA if a complete built-in test approach is required.

The main novelty and first goal of this paper is to present the first experimental characterization of this sensor topology, focusing on its sensitivity, noise and linearity. To this end, we implemented a realization of this sensor topology in a standard 0.35 μm CMOS technology (VDD = 3.3 V) together with MOS transistors acting as controllable dissipating devices. The second goal is to assess the temperature sensor for RF CUT monitoring. For that purpose, a tuned (420 MHz) class-A radio-frequency power amplifier (PA) is built-in together with the sensor, which shows its capability to monitor the PA central frequency and the output power delivered to the load (antenna).

Taking this into account, the paper is organized as follows: the sensor topology and design are described in Section 2. The sensor’s block characterization and full sensor validation are carried out in Section 3. Section 4 presents the PA used as CUT, the placement of the transducer within the PA layout and two application cases for this temperature sensor as a built-in RF monitor. Finally, Section 5 draws the main conclusions.

## 2. Sensor Description and Design

**Figure 2:**
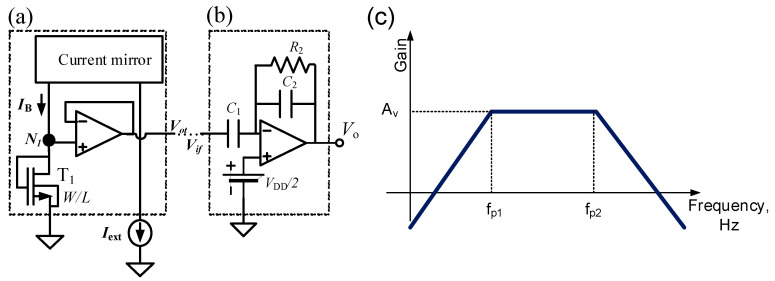
Temperature sensor schematic. (**a**) Temperature transducer based on an nMOS transistor (T_1_). (**b**) Active band pass filter (BPF). In our design: *I_ext_* is off-chip. (**c**) Typical BPF frequency response of the circuit in (**b**), characterized by the low-frequency (*f_p_*_1_) pole, the high-frequency (*f_p_*_2_) pole and the band-pass gain *A_v_*.

Figure 2 shows the proposed sensor schematic. It was made of two blocks: (a) the temperature transducer and (b) the active BPF. The goal of the temperature transducer is to generate a voltage at the node *V_ot_* proportional to the working temperature of the transistor T_1_, which is placed in the silicon die at the proximity of the CUT. On the other hand, the aim of the active BPF is to provide signal amplification at the output node *V_o_* if the input signal *V_if_* has its frequency within the passing band. The main figures of merit of the BPF frequency response are represented in Figure 2c: the low-frequency (*f_p_*_1_) and high-frequency (*f_p_*_2_) poles, which determine the pass-band, and the band-pass gain *A_v_*. Three different circuits were implemented in the IC: only (a) with *V_ot_* connected to an output pad; only (b) with *V_if_* connected to an input pad and (a) and (b) with *V_ot_* internally shorted to *V_if_*. A detailed description and theoretical analysis of each block is in the following two subsections, with emphasis on sensitivity, noise and linearity.

### 2.1. Temperature Transducer Description

The temperature transducer T_1_ was an nMOS transistor (dimensions: *W* = 24 μm, *L* = 1.5 μm) connected in diode configuration and biased with a DC constant current (*I_B_* in Figure 2). A small bias current placed the transistor in the weak inversion region, having an expected sensitivity of −1.5 mV/K at *I_B_* = 20 nA. With other dimensions and bias, transducer sensitivities in the range [−1.6 mV/K, 5 mV/K] can be achieved, as reported in [44,45]. To create such a small bias current, a current mirror with a ratio *I_B_/I_ext_* = 1/1000 was implemented. The internal current mirror in conjunction with an operational amplifier (OA) in voltage follower configuration ensures a low parasitic capacitance at node *N*_1_ and a low output impedance, enhancing the dynamic transducer behavior.

### 2.2. Band Pass Filter Amplifier: Noise and Linearity Analysis

The output of the temperature transducer was connected to an active BPF. The transducer signal is AC-coupled, amplified and filtered, and appeared at the output superposed on a DC level of *V_DD_*/2. Capacitors *C*_1_ = 50 pF and *C*_2_ = 100 fF set the band-pass gain *A_v_* which was designed to be:

(1) $$\left|A_{v}\right|≅\frac{C_{1}}{C_{2}}=500\;\left(54\;\mathrm{dB}\right).\;$$

Having a zero at the origin, the pass-band is determined by the low (*f_p_*_1_) and high (*f_p_*_2_) poles (cut-off frequencies), which are respectively:

(2) $$f_{p1}=\frac{1}{2\cdot \pi \cdot R_{2}C_{2}}$$

(3) $$f_{p2}=\frac{GBW}{\left|A_{v}\right|}$$

**Figure 3:**
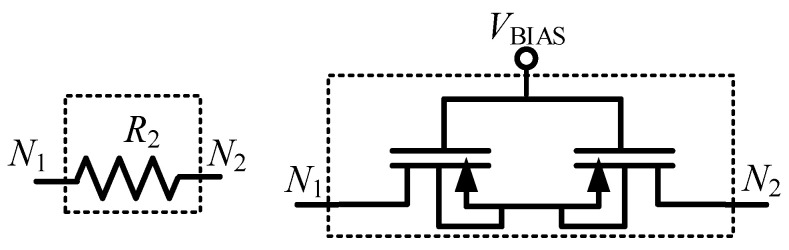
*R*_2_ implemented with two pMOS transistors biased in subthreshold.

Since the OA gain-bandwidth (GBW) product is 2.4 MHz, this results in a theoretical value for *f_p_*_2_ of 4.8 kHz. To have a low *f_p_*_1_, resistor values in the order of GΩ are required, thus *R*_2_ was implemented with two subthreshold biased pMOS, as shown in Figure 3 [46,47,48,49]. These MOS had *W* = 2 μm and *L* = 10 μm, and the external voltage *V_BIAS_* applied to their gate allows tuning to its equivalent resistance. From Equations (1) and (3), in a first order analysis, *f_p_*_2_ and *A_v_* should be independent from *V_BIAS_*.

Table 1 shows the values, obtained from simulations, of the *R*_2_ incremental resistance as a function of *V_BIAS_* (assuming both *R*_2_ terminals, *N*_1_ and *N**_2_***, at *V_DD_*/2) as well as the BPF’s low and high cut-off frequencies. Note how *f_p_*_1_ is highly sensitive to *V_BIAS_*, which opens a discussion on its optimum value. As explained above, transducer signal consisted of a tone at a frequency Δ*f*, which should fall within the filter passband. From the values in Table 1, Δ*f* around 1–2 kHz are reasonable choices. The filter output would be measured with a DMM that evaluates the total RMS voltage. Then, a narrow band-pass (high *f_p_*_1_) seems desirable in order to filter out as much noise as possible and reduce the measurement noise level. But, on the other hand, the amplified signal at the output node modulates the *R*_2_ value, thus producing distortion. The effect of this *R*_2_ nonlinearity on the overall sensor nonlinearity is relevant whenever *R*_2_ has a significant contribution to the signal path, i.e., when Δ*f* is around *f_p_*_1_ or lower. On the contrary, at Δ*f* values well above *f_p_*_1_, the effects of *R*_2_, including its nonlinearity, become negligible on the output linearity and gain. From this point of view, a low *f_p_*_1_ is desirable.

In order to assess the effect of the different *f_p_*_1_ choices on the sensor noise and linearity, Table 1 shows simulation results of the filter alone (no transducer included). Noise was evaluated after integrating the power spectral density at the filter output up to 100 kHz, while the maximum harmonic distortion (*THD_MAX_*) was also evaluated at the filter output obtained for a 1 kHz input sinusoid just before output clipping. Simulations show how linearity was dramatically degraded as *f_p_*_1_ was set near the signal frequency, while could be improved as *f_p_*_1_ is moved away from the signal. The price to pay was a moderate noise degradation. In view of these simulation results, *V_BIAS_* = 1.2 V (*f_p_*_1_ = 248 Hz) was selected as a good default setting, which can be increased (*f_p_*_1_ decreased) to produce a more linear response.

## 3. Sensor Implementation and Validation

The sensor was designed and manufactured in the CMOS 0.35 µm process. This microelectronic process provides four metal layers and two polysilicon layers. Capacitances *C*_1_ and *C*_2_ were implemented with polysilicon capacitors. The supply voltage (*V_DD_*) was 3.3 V (unipolar), and the IC was packaged in a QFN56. Its validation was performed first by each block standing alone and finally both connected, as detailed below.

### 3.1. Temperature Transducer

**Figure 4:**
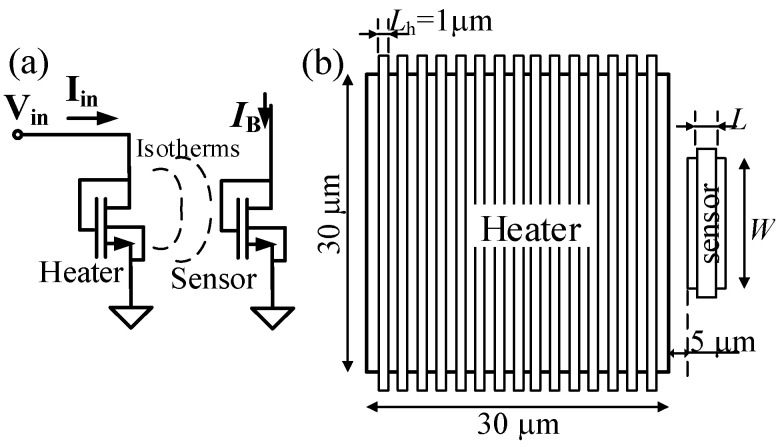
(**a**) Schematic of the nMOS heater and the temperature transducer T_1_. (**b**) Layout floorplan.

To characterize its frequency response, the circuit in Figure 2a was implemented as stand-alone, with the node *V_ot_* connected to an output pad. Besides, a large diode-connected nMOS transistor acting as a heater (*W* = 450 μm, *L* = 1 μm and 15 fingers) was placed at a 5 μm distance from the transducer (Figure 4). The heater was biased with a gate (drain) voltage, *v_in_*(*t*):

(4) $$V_{in}\left(t\right)=V_{DCh}+A\cdot \cos\left(2\pi \cdot f_{i}\cdot t\right),\;$$

where *V_DCh_* is the heater bias voltage, *A* is the AC amplitude, and *f_i_* is its frequency. Assuming a linear response, the current flowing through the heater is:

(5) $$I_{in}\left(t\right)=I_{DCh}+g_{m}A\cdot \cos\left(2\pi \cdot f_{i}\cdot t\right),\;$$

where *g_m_* is the heater AC transconductance at *V_in_* = *V_DCh_*, and *I_DCh_* is the DC drain to source current when *V_in_* = *V_DCh_*.

Multiplying Equations (4) and (5), the power dissipated by the heater in the frequency domain can be described as the sum of different spectral components. If we focus on the spectral component at the frequency *f_i_* (called *p_fi_*), it can be written as:

(6) $$p_{fi}\left(t\right)=P_{A}\cdot \cos\left(2\pi \cdot f_{i}\cdot t\right),\;$$

where *P_A_* is the power dissipation amplitude at the frequency *f_i_*. The above-mentioned heater dimensions enabled us to easily have *P_A_* in the mW range while applying a low input voltage. *p_fi_*(*t*) causes a temperature oscillation *t_S_*(*t*) at the temperature transducer with the same frequency *f_i_*:

(7) $$t_{S}\left(t\right)=T_{A}\cdot \cos\left(2\pi \cdot f_{i}\cdot t+\Phi_{A}\right),\;$$

where *T_A_* is the temperature amplitude and φ*_A_* is the phase shift between *t_s_* and *p_fi_*. Both thermal amplitude and phase shift depend on the silicon physical properties (thermal conductivity and specific heat), the input frequency *f_i_* and the distance between the heater and the transducer [40]. The close proximity between the heater and the transducer ensures a good thermal coupling even in the MHz frequency range [32,40,50,51]. Then, the transducer output voltage can be written as:

(8) $$V_{ot}\left(t\right)=V_{GST1}+V_{A}\cdot \cos\left(2\pi \cdot f_{i}\cdot t+\Phi_{A}\right),\;$$

where *V_GST_*_1_ is the DC gate-to-source voltage in T_1_, needed to sustain the drain current *I_B_*, and *V_A_* is the voltage amplitude generated by the oscillating temperature of amplitude *T_A_*. Here we assume that there is no phase shift between the transducer temperature and the output voltage, i.e., the pole induced by the node *N*_1_ in Figure 2b is at a frequency much higher than *f_i_*. Being a temperature-to-voltage transducer, its sensitivity is defined as *V_A_/T_A_*. However, as the goal of the sensor was to monitor the CUT characteristics through its power dissipation, the sensitivity expressed as *V_A_/P_A_* had a greater interest, and will be evaluated in this work.

**Figure 5:**
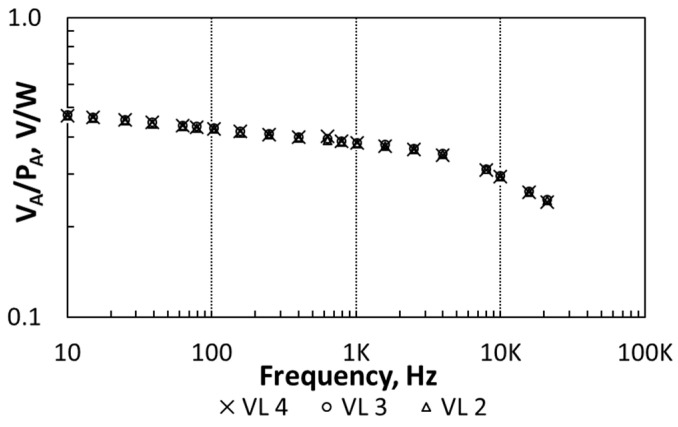
Measurements of transducer sensitivity as a function of the frequency. Three different voltage levels (VL) for the heating device are considered.

In order to characterize the transducer response, the heater has was excited with the different input voltage levels (VL) reported in Table 2. *P_A_* values in this table were calculated from the experimental I-V characteristics of the heater. Figure 5 shows the measured transducer sensitivity as a function of the frequency (transducer biased with *I_B_* = 20 nA). Here, the amplitude *V_A_* was measured with an LIA (Signal Recovery 7265DSP) locked at *f_i_* with a constant time of 1 s.

The frequency response in Figure 5 shows a low-pass behavior corresponding to the thermal coupling from the heater to the transducer, which agrees with the theoretical, simulations and experimental data previously reported in [52]. In this particular set-up, the coupling attenuation increased for frequencies higher than 3 kHz. Besides that, the independence of the transducer’s sensitivity on the amplitude *P_A_* proves the transducer linear behavior.

### 3.2. Band Pass Filter Amplifier

The circuit in Figure 2b was also implemented as stand-alone, with input (*V_if_*) and output nodes (*V_o_*) connected, respectively, to input and output pads.

**Figure 6:**
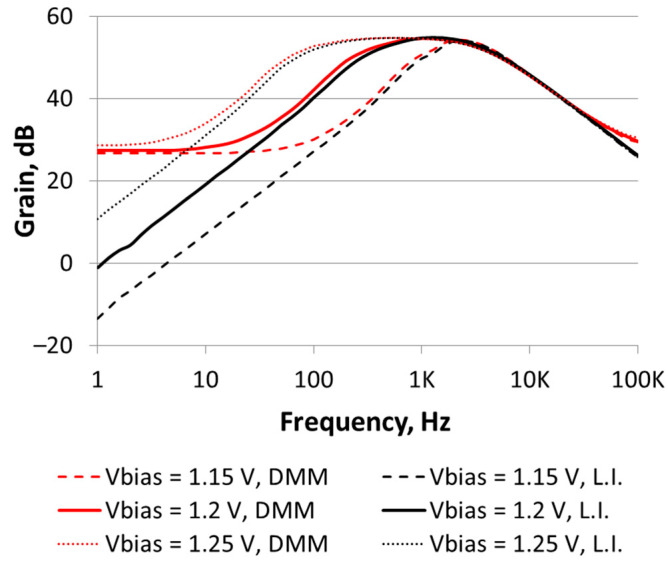
Frequency response of the amplifier BPF’s voltage gain, plot for three different *V_BIAS_* values: 1.15, 1.2 and 1.25 V. For each *V_BIAS_*, measurements are taken either with a digital multimeter (DMM) or with a lock-in amplifier (LIA).

Figure 6 shows the measured frequency response of the BPF voltage gain. Input *V_if_*(*t*) is a sinusoidal voltage of 20 mV_RMS_ attenuated by a factor of 100 with an off-chip resistive voltage divider. Filter characterizations were done for the three *V_BIAS_* values reported in Table 1. For each *V_BIAS_*, the output voltage amplitude was measured using either the LIA or a DMM (HP 33401A) AC-coupled. The LIA was set with a time constant of 1 s, which implied that the measure was integrating the signal (and noise) in a bandwidth of 1 Hz. On the other hand, the DMM was integrating the signal and noise along all the filter bandwidth (i.e., the output harmonics were integrated as well).

Table 3 summarizes the BPF characteristics extracted from the responses in Figure 6, considering LIA measurements. Comparing Table 1 and Table 3, measured characteristics show a reasonable agreement to the simulation predictions.

More importantly, measurements done with a simple DMM had very good agreement with those made with the LIA for frequencies equal and higher than the BPF central frequency. However, DMM results were higher than the LIA ones when the input signal frequency was equal or smaller than *f_p_*_1_. As predicted by simulations, output-voltage nonlinearities became relevant when the input frequency was equal or smaller than *f_p_*_1_, due to the *R*_2_ modulation. Those nonlinearities produced significant harmonics, which were captured with the DMM, thus producing a slightly higher gain measurement. Finally, DMM results showed an equivalent noise at the filter output of 4 mV_RMS_, which, according to Section 2, translated into an equivalent noise at the temperature sensor’s input of 7.54 mK (@ *I_B_* = 20 nA). As predicted by simulations, the noise level at the output was almost independent from *V_BIAS_*: *f_p_*_1_ may change over one order of magnitude, but its values were in the range of tens-hundreds of Hz, with small effect after integrating over the whole bandwidth.

**Figure 7:**
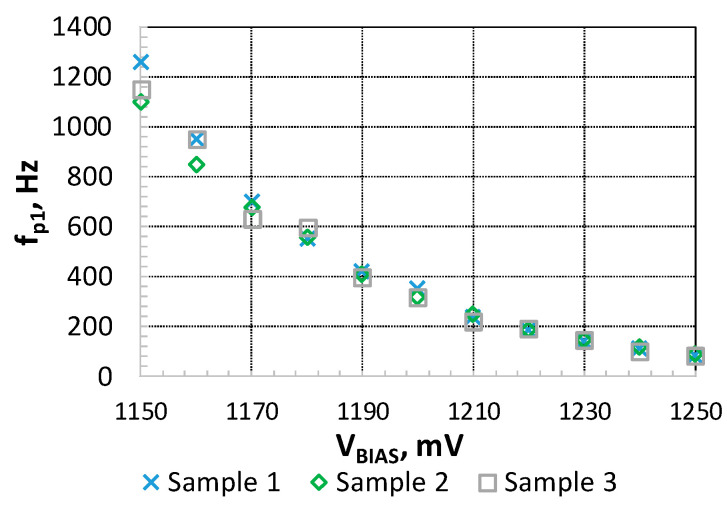
*f_p_*_1_ as a function of *V_BIAS_*. Measurements performed in three different samples.

The effects of the manufacturing variability on *R*_2_ were also evaluated. Figure 7 shows the effect of *R*_2_ sample-to-sample variability on the measured *f_p_*_1_ values, as a function of *V_BIAS_* in three different IC samples. In all these situations, the maximum gain measured was 54.5 ± 0.43 dB, in agreement with the expected gain independence with *V_BIAS_*.

### 3.3. Overall Sensor Characterization

Besides the stand-alone temperature transducer (Figure 2a) and the stand-alone BPF (Figure 2b), the complete circuit in Figure 2 was also implemented with the transducer’s output connected to the input of the BPF, and with a nMOS transistor such as that depicted in Figure 4 as a controllable heater. Then, when the dissipating device is biased with *v_in_(t)* as in Equation (4), the sensor’s output voltage can be written as:

(9) $$V_{O}\left(t\right)=\frac{V_{DD}}{2}+A_{O}\cdot \cos\left(2\pi \cdot f_{i}\cdot t+\Phi_{O}\right),\;$$

where *A_O_* is the amplitude of the sinusoidal component of *V_O_(t)* at the frequency *f_i_*. The filter introduces a phase shift (φ*_O_* − φ*_A_*) − φ*_A_* is defined in Equations (7) and (8).

**Figure 8:**
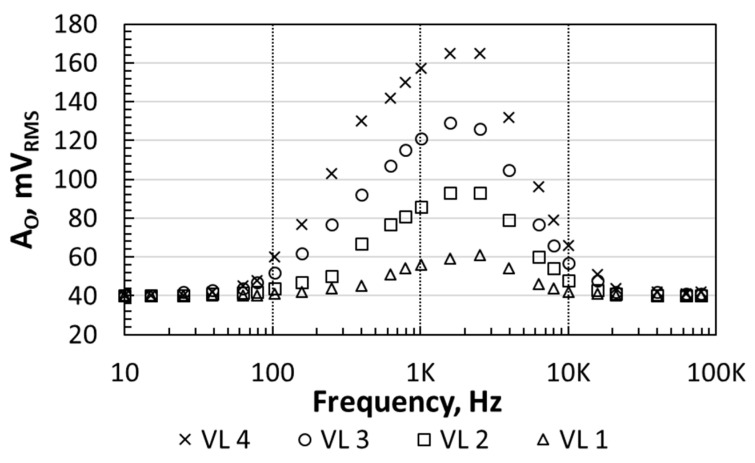
Sensor’s output voltage as a function of the frequency for four different heater bias. Measurements done with the DMM.

**Figure 9:**
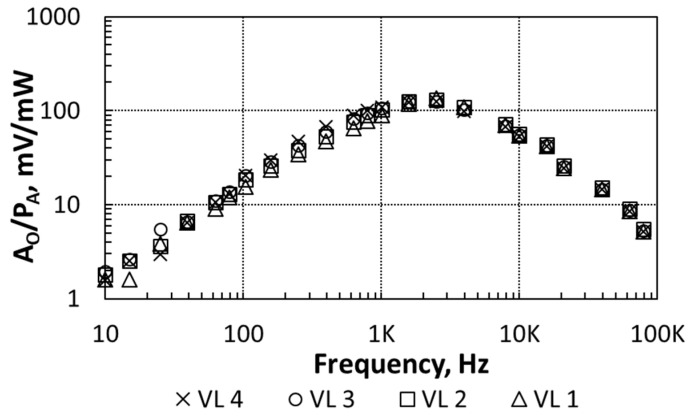
Sensor’s sensitivity as a function of the frequency. See Table 2 for the VL description and the PA values. Measurements taken with the LIA.

Figure 8 shows *A_O,RMS_* (RMS value of *A_O_* from Equation (9)) as a function of the frequency when measured with the DMM (@ *I_B_* = 20 nA, *V_BIAS_* = 1.2 V), using the different VLs in Table 2 for the heater excitation. The frequency response reported a central frequency, *f_c_*, of about 2 kHz for all the VL. The overall frequency response is the concatenation of the transducers’ and BPF frequency responses. For frequencies smaller than 100 Hz or higher than 10 kHz (the exact frequency depends on the particular bias), the sensor’s output voltage reached the noise level, which is 40 mV_RMS_. This noise level is higher than that observed in Figure 6, meaning that it was dominated by the heater-transducer circuits. The noise level sets the *P_A_* threshold that the sensor is able to detect. To calculate this threshold, Figure 9 shows the sensor’s sensitivity (*A_O_/P_A_*) as a function of the frequency, when measured with the LIA. The maximum sensitivity was about 140 mV/mW. Therefore, 40 mV_RMS_ corresponded to an equivalent noise floor in *P_A_* of 285 μW.

If we now consider the sensor’s linearity, the overlapping points in Figure 9 show that the sensor sensitivity was not affected by the *P_A_* level when *f_i_* was equal or higher than *f_c_*. On the other hand, the sensitivity depended on the amplitude *P_A_* dissipated by the heater when *f_i_* was smaller than *f_c_*: The *R_2_* nonlinear behavior affected the sensor linearity. These results have implications in the way the CUT must be driven when a test is done using a heterodyne excitation (Figure 1): Δ*f* should be higher than *f_p_*_1_. The exact value of Δ*f* is determined by the expected range of values of the amplitude *P_A_* dissipated by the CUT, which determines the required sensor sensitivity and the noise level. For instance, from Figure 8 and Figure 9, if Δ*f* = 2.5 kHz the sensor’s sensitivity is 134 mW/mV. This sensitivity allows the sensor to detect the *P_A_* dissipated by the heater when driven by the VL 1. On the other hand, if Δ*f* = 10 kHz the sensor’s sensitivity is 54 mW/mV, and the amplitude *P_A_* dissipated by the heater when driven by the VL 1 is below the noise level, whereas the sensor can barely detect the amplitude PA when driven by the VL 2. This indicates that Δ*f* higher than the sensor’s central frequency can be a good choice to sense large *P_A_* levels. On the other hand, if Δ*f* = *f_c_*, high values of *P_A_* might drive the sensor to saturation.

## 4. Use Case: RF Tuned Class-A Power Amplifier Monitoring

### 4.1. Circuit and Experimental Set Up Description

**Figure 10:**
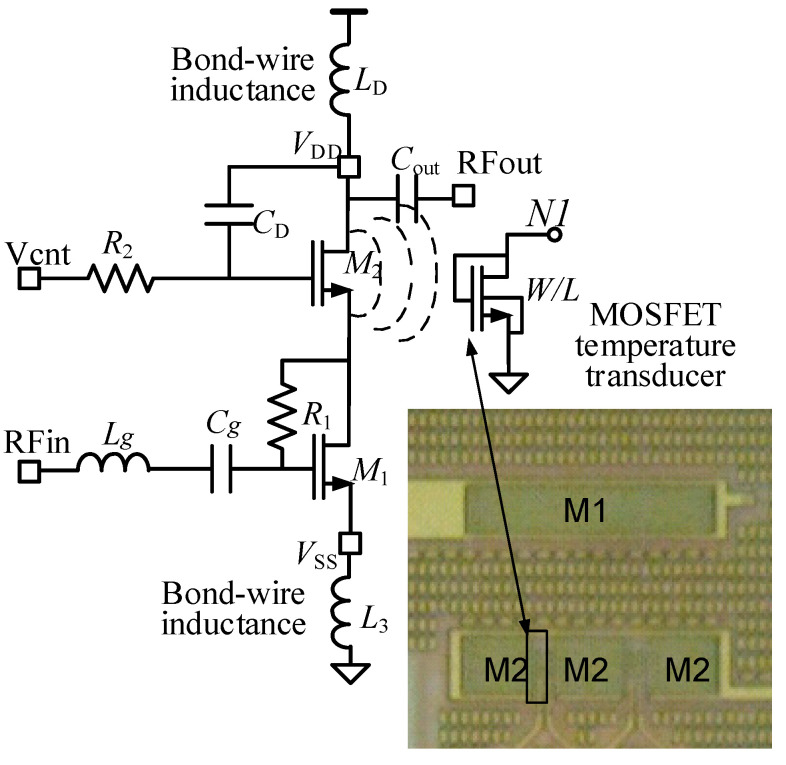
Schematic of the RF power amplifier used as CUT. The temperature transducer is placed close the cascode transistor M2. Inset shows a photo of the IC layout, with the placement of the active transistor M1, cascode transistor M2 (formed with three equal transistors connected in parallel), and the MOS acting as temperature transducer.

The BPF temperature sensor was integrated with a narrowband RF power amplifier (PA) used as a CUT. The schematic of the RF PA is shown in Figure 10. It is a class-A cascode amplifier with off-chip load, fully described in [32]. The cascode transistor *M_2_* was made of three transistors in parallel connection (inset in Figure 10). The overall *M_2_* dimensions were W = 1173 μm (implemented with 51 fingers), and L = 0.5 μm. The MOS temperature transducer was placed in the free space existing between two of these transistors. When the DC bias of the PA was *V_DD_* = 3.3 V and *V_cnt_* = 3 V, it had a current consumption of 22 mA. Experimental characterization of the PA reported a central frequency of 420 MHz, a maximum gain of 12 dB and a 1 dB compression point referred to the input of −4 dBm. To minimize parasitics in the RF measurements, the chip was directly soldered to the board (chip on board).

In order to demonstrate the sensor monitoring capability, the PA was AC driven with a heterodyne approach: two sinusoidal signals of input power *P_i_* and frequencies *(f_RF_ −* Δ*f*/2*)* and *(f_RF_ +* Δ*f*/2*)*. This driving generated power dissipation in transistor *M*_2_, with a spectral component at Δ*f* and an amplitude *P*_Δ*f*_. Previous work [32,33] indicated that *P*_Δ*f*_ depended on both the voltage gain of the amplifier at *f_RF_* and on the input-output matching at the same frequency. As elaborated in Section 3, this power dissipation generated an AC sensor output voltage superimposed to *V_DD_*/2, whose content at Δ*f* can be written as:

(10) $$v_{O}\left(t\right)=A_{O}\cdot \cos\left(2\pi \cdot f_{i}\cdot t+\Phi_{O}\right).\;$$

### 4.2. Output Power Monitoring

**Figure 11:**
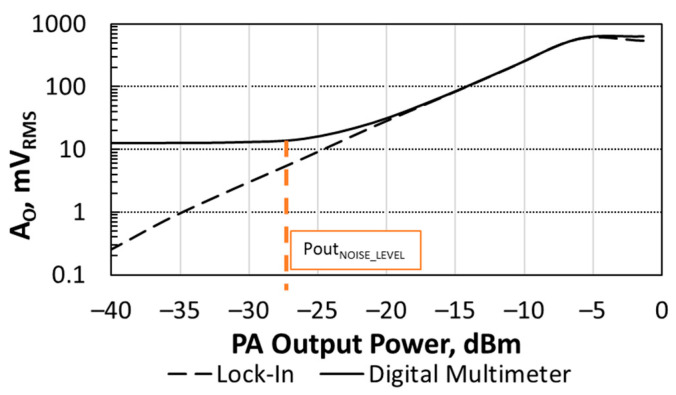
Sensors output voltage amplitude as a function of the output power delivered by the CUT to the load. Measurements obtained with a LIA and a DMM in AC mode. Label indicates at which *P_OUT_* the DMM readings reach the noise level.

Figure 11 shows the sensor’s output voltage amplitude *A_O_* as a function of the total output power delivered by the PA to a 50 Ω load. The temperature transducer was biased with a current *I_B_* = 20 nA and the sensor’s filter with *V_BIAS_* = 1.2 V. The PA was driven with two tones with *f_RF_* = 420 MHz (its central frequency) and Δ*f* = 1 kHz. *P_i_* was swept to get a total output power delivered to the load ranging from −40 dBm to 0 dBm. This output power range was below the 1 dB compression point, ensuring constant PA gain for all the cases. The PA output power was measured with the Agilent E4443A spectrum analyzer. The sensor output voltage was measured with both the DMM in AC mode, and the LIA locked at Δ*f.*

Focusing on DMM measurements, the sensor tracked the power delivered to the load when it was in the range [−25 dBm, −6 dBm] Below this range (−27 dBm, as labelled in Figure 11), DMM readings reached the noise level (which was 12 mV_RMS_,). At the other end of the sensor’s linear range above −6 dBm, the sensor output signal became clipped. LIA measurements show that the sensor was able to track the output power delivered to the load for values lower than −6 dBm, with very good agreement with DMM measurements in the range [−19 dBm, −6 dBm]. When the sensor output voltage became clipped, DMM measurements were slightly higher than LIA ones as DMM AC measurements take into account both the sensor’s output fundamental and the harmonics generated by the clipping.

If the sensor must track the PA output power for values higher than −6 dBm, the sensor sensitivity (*A_O_/P_A_*) should be reduced. For example, reducing *A*_O_/*P_A_* by a factor of ten would enable us to measure up to 4 dBm instead of −6 dBm. This would allow, for example, extending the sensor linear response up to the PA compression, and thus be able to monitor its 1-dB compression point.

**Figure 12:**
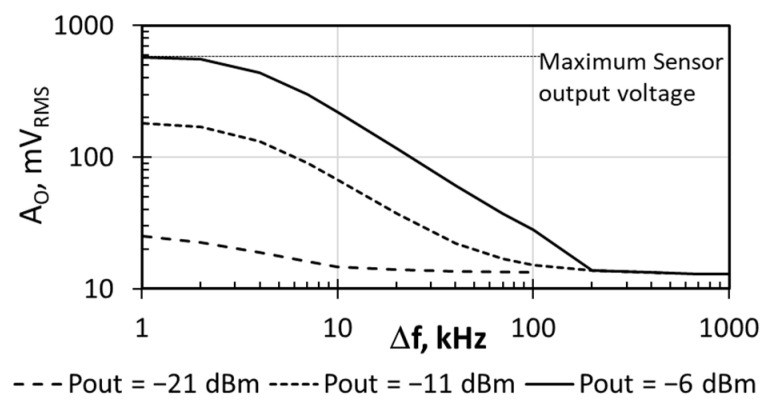
Sensor output voltage amplitude as a function of Δ*f* for three different output power levels. DMM measurements.

Several strategies can be followed to reduce the sensor sensitivity: (i) increasing the distance between the CUT and the temperature transducer T_1_ in the IC layout [40]; (ii) decreasing the BPF gain below the current *A_v_* = 500, which opens the possibility to implement BPF amplifiers with tunable gain for dynamic range extension and (iii) changing the transducer’s T_1_ dimensions or bias [43,44]. All these strategies require either a custom layout depending on the target measurements or the design of additional complex circuits, such as tunable gain networks or a programmable transducer bias. Nevertheless, there is another strategy that can be used without redesigning the sensor or the sensor placement. As pointed out by Figure 9 and discussed in Section 3, the sensor sensitivity can be reduced by choosing a Δ*f* value higher than *f_c_*. To illustrate this sensitivity reduction, Figure 12 shows the *A_O,RMS_* measured with the DMM when increasing Δ*f* above 1 kHz, for three constant PA output power values. Focusing on Pout = −6 dBm, with Δ*f* = 1 kHz the sensor output had already reached the saturation level observed in Figure 11. As Δ*f* was increased, the measured output amplitude decreased (i.e., the sensor enters in the linear range), thus enabling the possibility to monitor higher output powers. Finally, when Δ*f* = 200 kHz, the DMM output reached the noise level, and *P_out_* = −6 dBm became the Pout_NOISE_LEVEL_. From Figure 12, Pout_NOISE_LEVEL_ was −21 dBm when Δ*f* = 10 kHz, and −11 dBm when Δ*f* = 100 kHz. Therefore, Δ*f* selection allowed us to easily adjust the linear response of the sensor to different ranges of dissipated power.

### 4.3. Central Frequency Monitoring

**Figure 13:**
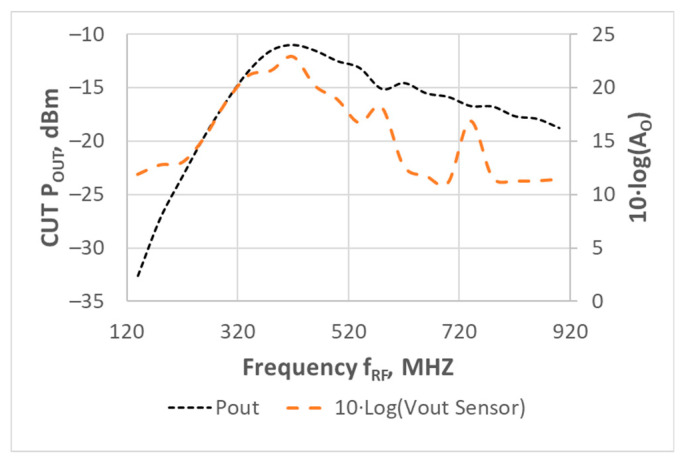
Sensor’s output measured with the DMM, superposed to power delivered to the load at the frequency *(f_RF_ +* Δ*f/2)* as a function of *f_RF_*. Δ*f* = 1 kHz, and total PA input power = −20 dBm (−23 dBm/tone). Sensor output voltage represented as in [33].

Figure 13 shows the RF power delivered to the load at the frequency *(f_RF_ +* Δ*f/2),* measured with the RF spectrum analyzer connected to the PA’s output; and the sensor’s output voltage measured with the DMM in AC mode; both as a function of *f_RF_.*

Sensor and PA had the same bias than the one reported in the previous section. In this experiment, the only swept input variable was the frequency *f_RF_* (from 120 to 920 MHz). In all the measurements, Δ*f* = 1 kHz, and the total PA input power was −20 dBm (−23 dBm each tone), i.e., the PA had a linear behavior. The spectrum analyzer measurements indicated that the PA central frequency was 420 MHz, which agreed with the frequency *f_RF_* where the sensor’s output amplitude at Δ*f* was maximum [31].

## 5. Conclusions

A single MOS transistor used as a temperature transducer connected to a BPF amplifier was presented, characterized and assessed for IC testing applications. The overall sensor circuit was implemented in a standard 0.35 µm CMOS technology, and was built-in with devices acting as controlled heat sources, and an RF power amplifier was used as a CUT. As strong points, heterodyne measurements could be done with a simple DMM, allowing a simplification of the measurement set-up. As the LIA was not required, there was no need for locking the frequencies of all the generators involved in the set-up measurements. Moreover, the task of the DMM could be easily integrated with the CUT, allowing a complete built-in self-test (BIST) solution. As weak point, as DMM integrated noise on a wider bandwidth, DMM readings did not reach the sensitivity levels achieved with an LIA. The sensor characterization showed that the nonlinear *R_2_* behavior did not affect the sensor’s linearity as long as Δ*f* was higher than the first BPF’s cut-off frequency. Moreover, the sensor sensitivity could be reduced by selecting a Δ*f* higher than the BPF’s central frequency, allowing extension of the sensor’s dynamic range. As a proof of concept, we showed the feasibility of the circuit to track the output power delivered to the load and the central frequency of a RF class-A power amplifier.

Future directions of our research are the usage of temperature sensors as monitors in circuits used to compensate the effects of time-variability (e.g., aging) in RF circuits.

## 6. Patents

PCT/ES2013/070095: Sensor Circuit for Obtaining small-signal temperature measurements in integrated circuits.

## Figures and Tables

**Table 1:** Active band pass filter (BPF) simulated characteristics.

*V_BIAS_* (V)	*R*_2_ (GΩ)	*f_p_*_1_ (Hz)	*f_p_*_2_ (kHz)	*A_v_max_* (dB)	Noise (μV^2^_RMS_)	THD*_max_* (dB)
1.15	1.47	973	5.5	54.8	2.04	−15.7
1.2	6.42	248	4.6	54.7	2.22	−29.9
1.25	28.5	55	4.6	54.5	2.46	−52.7

**Table 2:** Excitation applied to the heater.

VL	*V_DCh_* (V)	*A* (mV_RMS_)	*P*_A_ (mW)
1	1.67	7.8	0.44
2	1.67	15	0.84
3	1.67	23	1.29
4	1.67	31	1.75

**Table 3:** Active BPF measured characteristics.

*V_BIAS_* (V)	*f_p_*_1_ (Hz)	*f_p_*_2_ (kHz)	*A_v_max_* (dB)
1.15	1258	5.1	54.1
1.2	350	3.9	54.9
1.25	79	3.9	54.6

## Data Availability

Data sharing is not applicable to this article.
